# “I honestly didn’t know it was for women too…” acceptability and feasibility of integrating PrEP services into OB/GYN practices from the perspectives of practice staff and cisgender women in the community: a qualitative study

**DOI:** 10.3389/frhs.2025.1567688

**Published:** 2025-05-30

**Authors:** Christine M. Khosropour, Elise Healy, Emma M. Murphy, Arianna Rubin Means, Erykah Pasha, Jaelyn Howard, Emani Gonzalez, Tiffany D. Lloyd, Bethany Spier, Cathy J. Berry, Kandis V. Backus

**Affiliations:** ^1^ Department of Epidemiology, University of Washington, Seattle, WA, United States; ^2^ Department of Medicine, University of Washington, Seattle, WA, United States; ^3^ Department of Global Health, University of Washington, Seattle, WA, United States; ^4^ Layla’s Got You, Syracuse, NY, United States; ^5^ Cathy J. Berry and Associates, Syracuse, NY, United States; ^6^ Gilead Sciences, Rochester, NY, United States

**Keywords:** gynecology, obstetrics, HIV, healthcare delivery, pre-exposure prophylaxis (or PrEP)

## Abstract

**Background:**

HIV pre-exposure prophylaxis (PrEP) is effective at preventing HIV but uptake among cisgender women in the United States (US) is suboptimal. Most US cisgender women receive care in private practice settings, but PrEP has not been routinely integrated there. We investigated barriers and facilitators to integrating PrEP into women's health practices.

**Methods:**

In upstate New York in 2023, we conducted two focus group discussions (FGDs) with 22 cisgender women of color. Discussions focused on patient awareness/knowledge of PrEP, experiences accessing sexual healthcare, and preferences in services offered. We concurrently conducted one-on-one in-depth interviews (IDI) with 11 clinical staff (medical assistants, nurses, physicians/midwives) in an obstetrics/gynecology private practice. Interviews focused on staff awareness/knowledge of PrEP, desire to offer PrEP, and barriers/facilitators to integrating PrEP into practice flow. Thematic analysis, informed by the COM-B and Theoretical Domains Framework, was used to identify determinants of integration.

**Results:**

The median age of FGD participants was 20 and 72% identified as Black. Key themes included: low awareness of and misconceptions about PrEP (e.g., PrEP is for gay men); perceived stigma about PrEP (e.g., PrEP implies multiple sexual partnerships); previous negative experiences seeking medical care (e.g., feeling judged); desire for healthcare settings to integrate PrEP as part of holistic reproductive healthcare. Clinical staff had low awareness of and misconceptions about PrEP. Barriers to integrating PrEP included: lack of PrEP knowledge, lack of time to integrate PrEP services within routine visits, challenges with billing insurance for integrated services, and heterogeneity in comfort with sexual health discussions with patients. Facilitators included staff buy-in to provide PrEP and willingness to adapt clinical protocols to integrate PrEP, rooted in recognition that HIV prevention is important for their patients and community.

**Conclusions:**

Similar misconceptions about PrEP exist among cisgender women in the community and clinical providers in private practice, but both groups recognize the importance of expanding PrEP access. Despite high motivation to prescribe PrEP, there are unique structural barriers to integrating PrEP in the private practice setting (e.g., insurance and billing). Directly addressing shared and distinct patient and provider-level concerns may facilitate integration of PrEP services in private practices.

## 1 Introduction

HIV continues to be an important public health concern in the United States (US), with approximately 38,000 individuals diagnosed with HIV in 2022 (1). Cisgender women (individuals assigned female at birth who identify as women) account for approximately 20% of new HIV diagnoses, with Black cisgender women being disproportionately impacted (1). In 2022, Black women represented approximately 15% of the female population in the US but accounted for 47% of all new HIV infections among women (1). Although there were substantial declines in HIV diagnoses among women prior to 2020, there are still about 7,000 women newly diagnosed with HIV in the US each year; this number has remained stable for the past 5 years (2).

HIV pre-exposure prophylaxis (PrEP) is a medication taken by people who are at risk of acquiring HIV. PrEP is highly effective at preventing HIV, but uptake of PrEP has been markedly low among cisgender women in the US. In 2022, only 15% of cisgender women who could benefit from PrEP were prescribed it (3). There is a large body of literature describing barriers to PrEP uptake among women, namely inadequate awareness of PrEP, stigma, cost, and lack of PrEP access (4, 5). Another potential, but less well-described, driver of low PrEP uptake is the fact that there is often no clinical “home” for PrEP (i.e., no designated clinical specialty/sub-specialty or specialty setting to prescribe PrEP) (6–8). Family planning clinics are one location where PrEP services could be integrated into other care services accessed by cisgender women (9–11). However, about 80% of women in the US report going to a doctor's office (as opposed to a clinic or health center) for their healthcare (12), which underscores the need for PrEP provision to be integrated into primary care settings, such as women's health or obstetrician-gynecology (OB/GYN) offices.

There is general agreement that women's health and OB/GYN settings are an appropriate venue for PrEP provision (7, 13, 14); indeed, the American College of Obstetrics and Gynecology (ACOG) recommends that obstetrician-gynecologists discuss PrEP with all sexually-active patients and notes that PrEP can be prescribed by obstetrician-gynecologists and other allied health professionals (15). However, to date there has been little integration of routine PrEP provision into women's health or OB/GYN offices, and the facilitators and barriers to PrEP integration in these settings have not been well-described. Although clinical PrEP protocols (e.g., type of medication, what labs need to be drawn) are similar across clinical settings, operational protocols (e.g., billing) are not. Women's health and OB/GYN offices and private practices are operationally different than the clinical settings in which integration of PrEP has previously been emphasized (e.g., sexual health clinics); thus, a better understanding of the specific barriers to PrEP integration in these settings is critically important.

The objective of this qualitative study was to understand the perceived acceptability and feasibility of integrating PrEP services into an OB/GYN private practice in upstate New York, from the perspective of cisgender women in the community and OB/GYN practice staff at a health facility launching PrEP for the first time.

## 2 Materials and methods

### 2.1 Study population

The study was conducted in upstate New York (NY), in a county with disproportionately high sexually transmitted infection (STI) rates relative to the rest of the state, excluding NY City. In April 2023 we invited women of color aged 18–26 living in upstate NY who access reproductive health services to participate in one of two focus group discussions (FGDs). To identify eligible FGD participants, we collaborated with Layla's Got You, a community-based organization that aims to reduce unintended pregnancies and advocate against barriers that impact women's reproductive health. The Layla team, which is led by Black and Brown women, promoted the FGDs across social media platforms and via word-of-mouth to their social networks. Individuals who were interested in participating were referred to the study team. A maximum of 15 participants were invited to each FGD.

To gain the perspective of practice staff, we worked with an OB/GYN private practice in upstate NY to identify participants for in-depth interviews (IDIs). The practice has two office locations (one urban and one peri-urban) and has over 20,000 patient encounters annually; approximately one-quarter of encounters are with women of color. We purposively sampled interviewees based on staff role, and aimed to interview people at both office locations. All staff were eligible to participate in the interviews. Our goal was to interview at least one staff member in each role (e.g., administrative staff, physician or advance practice provider, medical assistant, nurses). Interviews were conducted in May-June 2023.

### 2.2 Models and frameworks

We used the Capability, Opportunity, Motivation-Behavior (COM-B) Model (16) and Theoretical Domains Framework (TDF) (17) in this study. The COM-B is a behavior change framework that attempts to understand how an individual's capability (C), opportunity (O) and motivation (M) affect behavior change and underlying determinants of organizational change. The TDF includes a further 14 domains that can be mapped to the COM-B model, providing a more granular understanding of the barriers and facilitators to behavioral change (18). We drew from all 14 TDF domains which we hypothesized to affect adoption and implementation of integrated PrEP services. These domains were used to develop a semi-structured interview guide and initial deductive codebook, as described below.

### 2.3 Data collection

Study procedures were approved by the University of Washington Human Subjects Division. All participants provided verbal consent prior to data collection.

FGDs took place in person. At the beginning of each FGD, we asked participants to complete an anonymous paper survey which queried them on demographic characteristics (e.g., age, race) and asked a series of questions to ascertain their knowledge of HIV and PrEP. Participants were compensated $50 for participating in the FGD. IDIs were conducted via Zoom and participants were compensated $25 for participating in an interview.

The FGDs and IDIs were both guided by a semi-structured interview guide informed by the COM-B and TDF. The interview guides were adapted from COM-B and TDF standardized question guides and adapted from interview guides used in a previous study of PrEP uptake (19). Both guides were reviewed by individuals belonging to the same community as the FGD/IDI participants.

FGD and IDI were led by study team members trained in facilitation and interviewing (FGD led by EH and KVB; IDI led by CMK and EMM). In both FGDs and IDIs, participants were asked about their familiarity with PrEP, the importance of PrEP in their community or the community they serve, and how PrEP could best be implemented in a primary care setting. In addition to these topics, IDI participants were also asked about potential structural barriers and facilitators to integrating PrEP in their practice. FGDs and IDIs were audio recorded and professionally transcribed. The interviewers completed structured debrief notes after each interview, and transcripts were reviewed for quality and fidelity to the audio files prior to uploading transcripts into Dedoose (20) for coding.

### 2.4 Analysis

We used a mix of deductive and inductive thematic coding approaches. The initial deductive codebook was developed based on select constructs from the COM-B Model and the TDF. Additional inductive codes were developed upon reviewing the transcripts, based on emerging themes not captured by the COM-B and TDF codes.

Transcripts were coded by two trained qualitative analysts (EH and EMM). Each transcript was coded by a single primary coder; coded transcripts were then reviewed and validated by a second coder. Coding started with both coders primary-coding a single transcript and meeting to discuss differences in applications of codes. Codebook adjustments were made after this initial coding before the coders preceded with the remaining transcripts. Inter-coder reliability meetings were conducted throughout the analysis period to ensure similar applications of codes and to make codebook edits as new inductive codes emerged. A standardized validation tracker was used to record instances of disagreement between coders and note final coding decisions; a third researcher served as a tiebreaker as necessary (ARM). After all transcripts were coded, thematic memos were developed (prepared by EH and reviewed by CMK, KVB, ARM, EMM), organized by each of the COM-B components and relevant TDF domains. Key themes were derived from analysis of these memos, by exploring patterns and relationships between COM-B components and TDF domains. The project team then presented these key themes to practice staff and a sub-set of FGD participants during two member-checking meetings to validate findings. Members endorsed these themes, and no additional changes were made following member-checking.

## 3 Results

We conducted two FGDs that each lasted for 90-minutes. The FGDs comprised 9 and 13 individuals in each. Participants were on average 20 years old, most (72%) reported being Black race, nearly all (91%) had heard of HIV or AIDS but less than half (48%) had heard about PrEP (Table 1). Eleven staff at the practice participated in IDIs, including 2 administrative/operations staff and 9 clinical staff (physicians, advanced practice providers, midwives, medical assistants, and nursing staff). Most staff had been at the practice for >3 years. In contrast to the FGD participants, practice staff were given some background about PrEP as part of the project introduction prior to being interviewed, though the majority (82%) stated in their interview that they had at least heard of PrEP prior to the start of the project. IDIs lasted between 20 and 40 minutes each.

**Table 1:** Characteristics and HIV/PrEP knowledge of focus group participants (*N* = 22).

Characteristic	*N* (%)
Age in years, median (IQR)	20 (19–23)
Race	
Black	16 (72.3)
Black and White	3 (13.6)
Black, White, American Indian	1 (4.5)
Asian and Pacific Islander	1 (4.5)
Not reported	1 (4.5)
Hispanic ethnicity	3 (13.6)
Ever heard of HIV or AIDS	20 (90.9)
Ever heard of PrEP	10 (47.6)
“How likely do you think you are to get HIV in your lifetime?”	
Not at all likely	8 (36.4)
A little bit likely	9 (40.9)
Somewhat likely	5 (22.7)
Very likely	0 (0.0)
“Do you believe that women in your community are at risk of getting HIV?”	
Yes	21 (95.5)
No	1 (4.5)

IQR, interquartile range.

Major themes from the FGDs and IDIs converged. These themes include: (1) there is awareness of PrEP's potential appropriateness amongst all participants but a lack of understanding about which populations could benefit from PrEP; (2) opportunities to offer PrEP are compromised by lack of comfort between patients and providers; and (3) despite staff's willingness to offer PrEP, the practice's ability to integrate PrEP into services is limited by structural barriers such as time and billing.

### 3.1 Theme 1: There is awareness of PrEP as a potentially appropriate intervention, yet a lack of clarity about who could benefit from PrEP (TDF domains: knowledge, environmental culture, intention, and optimism)

Although participants rarely stated that HIV was common in their community, both FGD and IDI participants reported that STIs, including HIV, were a common concern among young women in their community. FGD participants discussed infidelity of male partners in their community, a discomfort talking about sexual health with sexual partners, and noted that many people are not regularly tested for STIs. Practice staff similarly reported that their younger patients frequently came in for STI testing, often due to concerns about their partner's sexual behaviors.

These are 20-year-old, 24-year-old girls that still will be like “STDs, I don't want to talk about that.”. If you don't talk about it, you don't talk about getting yourself tested.one of the reasons we don't talk about it because there's so much taboo around the discussion. I think shame is a big factor, especially for black women.

FGD 1

As far as that goes, I would say, a lot of them have concern about infidelity and where their partner's been and they don't know. We do get a fair amount of patients that come in with those concerns. Whether they call specifically to come in for that or they're for something else and it comes up in topic then yes. I hear that [STI concerns] a lot.

-IDI 2

I feel like in our demographic of patients. We do get a lot of patients concerned with STDs, just STDs in general. We get a lot of younger girls who come in for STD testing. That's a pretty big thing I would say that we deal with.

-IDI 7

Most IDI and FGD participants had heard of PrEP prior to data collection, but few were aware of the specific indications for prescribing PrEP. Specifically, many IDI and FGD participants believed that PrEP was exclusively for men who have sex with men (MSM).

I can't remember which one is which, but I know there's one [medication] that you take if you already have HIV, and it helps to keep the count low. Then there's the [medication] where if you think you have been exposed to HIV, you take it, then they monitor you to make sure you don't catch it.

-FGD 2

They were like, “PrEP is for anybody who is engaging in anal sex, so if you're doing that, take PrEP.” I'm like, “Oh, I'm not, but thanks for that.”

-FGD 1

I didn't know much about it and I still really don't know much about it other than I had seen some commercials on TV about the HIV prEP and it did seem more geared towards I felt like the male population, the gay population in the commercial. I honestly didn't know it was for women too

-IDI 1

Upon further clarification from the study team about PrEP eligibility, both IDI and FGD participants suggested that PrEP could benefit women in their community. Despite suggesting that PrEP makes sense for young women of color, FGD participants often said that they themselves would likely not take PrEP though many indicated that they knew people in their community who would be interested.

I know for me personally, right now, I'm [sexually] abstinent..but I feel like..for somebody who's about to [sexually] engage with somebody they don't know or they haven't known them that long. Then that's essentially a good demographic to try and encourage it, but yes, people can be real trash, and they can get a fake like, “Oh, yes, like I'm clean” and they're not..

-FGD 1

I think it's important. I don't know how big the need is because most of our patients that I know of that have been tested [HIV] negative, but I definitely know that there is a lot of our population of patients that are sexually active with multiple partners and don't use protection, so I would say yes, it would be a beneficial thing to have for our patients.

-IDI 7

### 3.2 Theme 2: Opportunities to offer PrEP are compromised by lack of comfort between patients and providers (TDF domains: environmental culture, social influences, and beliefs about capabilities)

Both IDI and FGD participants suggested that sex and sexual health were often culturally taboo topics. Furthermore, IDI and FGD participants frequently used potentially stigmatizing language to describe women who could benefit from PrEP, likening PrEP eligibility to “promiscuity”.

I've never heard the types of PrEP. I've only ever heard “PrEP”. Nobody goes into detail about what PrEP is necessarily. And I also feel like there's stigma where people think that you have to have sex with a lot of people and not just one person.

-FGD 1

Well, we are like a GYN office and there are people that are promiscuous and you never know.

-IDI 6

While many IDI participants said they were comfortable discussing sexual health/behavior with patients, FGD participants described experiences in other healthcare settings where they often felt judged or belittled by clinical staff and recounted instances that providers did not believe them or acted like they understood a patient's body better than the patient. Numerous FGD participants provided examples from other healthcare settings of moments when they felt disregarded and/or disrespected during a healthcare visit. These experiences contribute to a general lack of demand for sexual health services, including PrEP.

Not only should the doctors and the nurses and the people working the desk, the person who is the receptionist, the person who is coming to get you, the person who is coming to give you the medication, all these people should not be judgmental. They need to be able to talk to you properly. They need to be able to understand… yes there's a lack of respect from a lot of these nurses. They put their personal feelings in when people come in. People don't wanna come and get tested. People don't wanna come and get birth control. People don't wanna come and get abortions.

-FGD 1

If you come to them with concerns and I, for example, I thought I had PCOS, and I had very irregular periods. So I went to the doctor, he's like, “You're fine, you've only had you're period since you were 12 and you're 17, you're fine.” That's five years, it should have regulated itself by now. But when you're told by a professional, especially someone who you trust,..you think they know what they're talking about, and they dismiss you so quickly, you start to question yourself, “Maybe I'm overreacting, let me just ignore it,” or they're obviously the professional, they have 40 years of experience, so you're like, “I'm fine.”

-FGD 2

There were some practice staff who acknowledged that they could tell that some patients felt judged or misunderstood during their visits.

I think there probably would have to be a bigger conversation of, we are not judging your choices, we are not judging who you are in your sexual practices, we just want to keep you and those around you as safe as possible. I think feeling judged is largely where people are hesitant in telling us their full history.

-IDI 9.

### 3.3 Theme 3: Despite staff's willingness to offer PrEP, the practice's ability to integrate PrEP into services is limited by time and billing (TDF domains: goals, intention, and environmental context)

Staff were overwhelmingly in favor of integrating PrEP into the practice's existing services. All practice staff interviewed, regardless of staff role, supported the idea of offering PrEP to at least some patients. However, there were mixed responses from staff about how PrEP services would be operationalized. Some respondents felt that PrEP could be easily integrated into routine visits but many reported tight schedules that would not accommodate additional PrEP counseling.

For me, my schedule might get a little busier, but it's just a couple of minutes, so it's not that big of a deal to me.

-IDI 1

I'm also concerned...because we are down providers of how we feasibly are going to do this because we don't have many providers at this point to be able to do it. Where are we going to fit it in the schedule on top of the OBs and other GYN patients?

-IDI 9

Additionally, there were external pressures related to insurance coverage and visit billing that would impact how PrEP could be integrated into the practice. Many practice staff stated that they were unsure if they could “add on” PrEP consultation to other routine visits because they may not be able to bill insurance for it. For example, initial PrEP visits could likely not be on the same day as a yearly exam because most insurance companies would not allow the practice to bill for two visits on the same day, forcing a PrEP initiation visit to be held at a later date.

I wonder like if I was to have somebody coming in for their yearly exam, there's only certain things I'm allowed to add on to that because of insurance purposes. I can't add in a biopsy if it's necessary or a fertility conversation. I can't add that on but I don't know how it would work for insurance purposes to add on also a PrEP conversation, if that's even allowed to be doing as a combined appointment.

-IDI 9

This presents a potential issue for young women seeking PrEP, as many FGD participants stated that they wanted more integrated care and an ability to have one healthcare visit address multiple concerns.

I think they should really lump them together. You're supposed to go to the doctor's every couple weeks or months or whatever it is. All of this should be encompassed together because it's all a part of my life. The same way I'm going to get my breast checked, or whatever, it should all be together because at the end of the day it's all about my overall health. I think that would help with the maintaining of the appointment or the maintaining of the process.

-FGD 1

## 4 Discussion

In this qualitative study, we aimed to investigate barriers and facilitators to integrating PrEP into women's health practices from the perspective of cisgender women in the community and staff at an OB/GYN private practice. We found that participants knew about PrEP, but among women in the community and practice staff there were misconceptions about who could benefit from PrEP. We also found that discomfort around sexual health discussion and judgement could limit opportunities to offer and uptake PrEP. Despite this, participants identified a need to, and enthusiasm about, integrating PrEP into women's healthcare services. Importantly, our findings identified key barriers to implementing PrEP into private practice settings, such as insurance and billing, which could limit the integration of PrEP provision in these settings. Our study suggests that strategies to integrate PrEP provision will likely need to be tailored to private practice settings.

### 4.1 Comparison with existing research

Our findings related to barriers to PrEP uptake and desire for integrated PrEP services largely align with those of other studies. Inadequate awareness of PrEP and stigma are noted barriers to PrEP uptake among cisgender women in the US (4, 5). In this study, we similarly found that most participants were unaware that PrEP could be used by women. We also noted that women in the community and practice staff mentioned discomfort with sexual health discussions which could create stigmatizing environments. However, we also found that women in the community were enthusiastic about the potential to have all sexual health services integrated into spaces where they already receive care. This finding adds to the growing body of literature supporting integration of PrEP into primary care and OB/GYN settings. In a large study of cisgender women in New York City, nearly 92% of respondents noted that primary care providers and OB/GYN were participants' preferred sources for PrEP (21). In a qualitative study by Danvers and colleagues, participants felt that OB/GYNs were experts in sexual and reproductive healthcare and that existing trust between a patient and their OB/GYN is a facilitator for women to consider PrEP (22). Together, these findings suggest that OB/GYN settings are first and foremost important venues to provide education to patients about PrEP. But that they also play an important role in facilitating PrEP uptake by integrating PrEP provision into routine reproductive and sexual healthcare.

### 4.2 Implications for policies and practice

We conceptualized this study as a formative step to integrate PrEP services into an OB/GYN private practice. The main themes from this qualitative study highlight three tangible steps to optimize integration of PrEP into private practice settings.

First, there is a need for targeted and continuing education about PrEP for clinical staff that provide women's health services. Although ACOG explicitly recommends that OB/GYN discuss PrEP with all sexually-active patients (15), our interviews revealed that most staff had only heard about PrEP through informal channels, such as lay advertisements, and many thought PrEP was only for gay men. In busy OB/GYN practices with innumerable competing demands, ongoing provider and staff education can be challenging, particularly for conditions that are not routinely encountered (e.g., HIV). Furthermore, since educational needs differ across staff roles at OB/GYN practices (e.g., provider, medical assistant, administrative staff) tailoring ongoing training can be challenging. Nonetheless, leveraging existing resources for provider education, such as national online curricula (23) or AIDS Education and Training Centers (24), may be an efficient way to keep practice staff up-to-date with contemporary recommendations.

Second, universal “screening” for PrEP may be an optimal way to integrate PrEP services into an OB/GYN practice. In interviews and focus group discussions, participants discussed how conversations between patients and providers about sexual health can be uncomfortable and leave patients feeling judged and stigmatized. Normalizing PrEP by introducing PrEP to all patients—without needing to ascertain information on sexual history—can help overcome this barrier. This approach has been noted by other studies ascertaining women's acceptability of receiving PrEP by their OB/GYN (22) and is the approach recommended by ACOG (15). Building new automated prompts, or stop actions, in the electronic medical records systems may be one way to support clinical staff in implementing new screenings and services with consistency (25). Motivated by the findings from this study, we have worked with the OB/GYN practice to begin including a PrEP information sheet as part of a patient's intake paperwork. The sheet provides an overview of PrEP and gives patients the option to indicate whether or not they would like to speak with their provider about PrEP. This approach allows patients time to learn and reflect about PrEP privately before being face-to-face with a provider. It also minimizes the need for an “interrogation” of a patient's sexual practices in order to introduce PrEP as an HIV prevention tool.

Third and importantly, the integration of PrEP into private practice can be a logistical challenge without a “one-size-fits-all” solution. Nearly all literature to date has focused on the clinical aspects of integrating PrEP (e.g., drawing labs, etc.) (13); very few have discussed operational issues such as insurance and billing. Our interviews with practice staff revealed that insurance and billing were primary drivers for how PrEP could be integrated into the practice. Specifically, initial PrEP visits could likely not be on the same day as a yearly exam because it would not be covered by most insurance. This runs counter to the desires of focus group participants who explicitly mentioned the desire to have integrated services that occur within a single visit. These competing scenarios are more common in private practice settings, where insurance and billing are often, by necessity, at the forefront of a practice's operations. There is a need to create and disseminate models of PrEP provision within private practice settings that are responsive to patient's needs but are also fiscally responsible (for both the patient and the practice). If such models exist, more widespread dissemination of these types of protocols and associated resources could be used as blueprints for other practices for whom the logistical hurdles to provide PrEP are daunting.

### 4.3 Strengths and limitations

Our study is strengthened by the inclusion of potential consumers of PrEP (women in the community) and providers of PrEP (staff at a clinical practice). Understanding the perspectives of staff in a private OB/GYN practice provides unique insights into how PrEP could be integrated into services where women usually seek healthcare. Limitations of our study include potential lack of generalizability to community members and practices outside of upstate NY. Additionally, during the FGDs, participants were not specifically queried about receiving PrEP at a private practice or OB/GYN office, but rather about integration of PrEP into other healthcare services generally.

## 5 Conclusion

In summary, despite the approval of PrEP for HIV prevention in the US in 2012, integration of PrEP into settings where cisgender women normally seek care has not been fully realized. We found that staff in an OB/GYN private practice were willing and enthusiastic to integrate PrEP into their services, and that cisgender women in the community prefer a “one-stop-shop” for their sexual and reproductive healthcare. At the same time, private practice offices have unique barriers to PrEP integration related to insurance and billing that may make integration a challenge. Future research could aim to identify and optimize strategies for PrEP integration and uptake to help elucidate models of PrEP integration specifically for private practice settings. Strategies such as same-day PrEP start, task-shifting, patient navigation, and provider incentives may be appropriate, but need to be tested and likely tailored for the private practice environment. This type of rigorous implementation research could facilitate PrEP integration for cisgender women which could ultimately improve PrEP uptake and decrease new HIV transmissions.

## Data Availability

The case memo data supporting the conclusions of this article will be made available by the authors, without undue reservation.
